# The Influence of Gender, Age, Matriline and Hierarchical Rank on Individual Social Position, Role and Interactional Patterns in *Macaca sylvanus* at ‘La Forêt des Singes’: A Multilevel Social Network Approach

**DOI:** 10.3389/fpsyg.2016.00529

**Published:** 2016-04-18

**Authors:** Sebastian Sosa

**Affiliations:** Formerly affiliated with Grupo de Conducta Adaptativa e Interacción, Psychology Faculty, University of BarcelonaBarcelona, Spain

**Keywords:** social network analysis, multilevel analysis, non-human primate, allogrooming, antagonism, individual attributes, homophily

## Abstract

A society is a complex system composed of individuals that can be characterized by their own attributes that influence their behaviors. In this study, a specific analytical protocol based on social network analysis was adopted to investigate the influence of four attributes (gender, age, matriline, and hierarchical rank) on affiliative (allogrooming) and agonistic networks in a non-human primate species, *Macaca sylvanus*, at the park La Forêt des Singes in France. The results show significant differences with respect to the position (i.e., centric, peripheral) and role (i.e., implication in the network cohesiveness) of an individual within a social network and hence interactional patterns. Females are more central, more active, and have a denser ego network in the affiliative social network tan males; thus, they contribute in a greater way to the cohesive structure of the network. High-ranking individuals are likely to receive fewer agonistic behaviors than low-ranking individuals, and high-ranking females receive more allogrooming. I also observe homophily for affiliative interactions regarding all attributes and homophily for agonistic interactions regarding gender and age. Revealing the positions, the roles, and the interactional behavioral patterns of individuals can help understand the mechanisms that shape the overall structure of a social network.

## Introduction

Animal societies are complex systems in which individuals have non-random and complex interactions, and are likely to develop behavioral strategies (Dunbar, 1989). This leads to the formation of a multilayered and multi-behavioral structure. However, questions persist about the fundamental evolutionary process by which a society emerges, stabilizes, and adapts.

Previous studies of animal species, including human and non-human primates, have investigated the behavioral differences and interactions among individuals according to attributes such as gender (Fedigan, 1982), age (Wey and Blumstein, 2010), body size (Archie et al., 2006), social status (Bergman and Moore, 2003), reproductive state (Cavigelli and Pereira, 2000), and kinship (Widdig et al., 2001). This study focuses on four specific attributes: gender, age, matriline (matrilineal kinship), and hierarchical rank.

Differences in gender lead to contrasting reproductive (Fragaszy and Mitchell, 1974; Fedigan and Baxter, 1984; Pereira, 1988; Cords, 2002) and behavioral strategies (Fedigan, 1982), and in particular, the expression of aggressiveness and allogrooming.

Age, or more precisely, ontogenesis (i.e., the development of an organism), influences the evolution and development of social relations and species-specific behaviors that are largely affected by interactional experiences with congeners (Harlow and Suomi, 1974; Hinde, 1974; Wilson, 1980; Shimada and Sueur, 2014). For example, hierarchical rank acquisition appears to be closely related to age (Borries et al., 1991) and the early experiences of juveniles (Mitchell et al., 1967; Olds et al., 1997). However, this influence differs according to species and gender (Sosa, 2015). Additionally, older individuals and females in particular are more likely to experience social exclusion (i.e., decrease in social interactions) (Hauser and Tyrrell, 1984).

One major kinship phenomenon among the animal kingdom is the matrilineal rank inheritance (MRI) (Kawamura, 1958) observed in macaques. It consists of the transmission of hierarchical rank from mother to daughter; the latter acquires the hierarchical rank directly below that of her mother. In addition, as according to the youngest ascendancy rule, young females outrank their older sisters (Thierry et al., 2004). The MRI process is made possible by nepotism, in that related females support each other during conflicts against non-kin females and help juvenile females outrank their older sisters (Cheney, 1977; Datta, 1983; Chapais and Gauthier, 1993). Furthermore, an adult female can outrank her mother when she is old and subsequently lacks kin support and has limited physical ability (Chapais and Berman, 2004). See Chapais and Berman (2004) and Hepper (2005) for an overview.

Social network analysis (SNA) is one approach used to analyze systems (Sueur et al., 2011) as complex as animal societies. SNA was first applied in psychological studies and, for a few decades, in animal social research (see Prell, 2011 and Brent et al., 2011 for an overview of SNA epistemology). However, certain methodological precautions must be taken when using any of the various analytical techniques based on SNA (Wasserman and Faust, 1994; Krause et al., 2009; Brent et al., 2011). In this study, I describe an analytical protocol based on SNA tools that compensates for the intrinsic limitations of animal behavioral data (i.e., dependency of data) and allows the analysis of weighted networks (network with weighted links).

Several studies have used SNA tools to examine the position and role of group members in non-human primates and other animal species. Lusseau and Newman (2004) revealed that central individuals are key players in maintaining social cohesion and have greater knowledge of their environment. In some non-human primate societies, central individuals are high-ranking animals (Kanngiesser et al., 2011). Using an interspecific comparative approach, several studies have analyzed network metric variations and succeeded in linking them to variability in social structure and dominance style (Sade, 1972; Voelkl and Noe, 2008; Sueur et al., 2011). Previous studies have also found that individuals from the philopatric gender are more central within a network (Smuts, 1985; Matsuda et al., 2012). In this way, central individuals play an important role in group cohesion and their position depends on several individual characteristics. Thus, identifying these central individuals according to their attributes could allow us to better understand how a social structure is shaped.

SNA research also addresses the principles of homophily and heterophily that refer to preferential interactions between similar (homophily) or dissimilar (heterophily) individuals (Lazarsfeld and Merton, 1954). These phenomena have been observed in many animal species: cetaceans (Lusseau and Newman, 2004), fishes (Croft et al., 2005), marmots (Wey and Blumstein, 2010), and human (McPherson et al., 2001) and non-human primates (Silk, 2001; Cords, 2002; Carter et al., 2015). However, animal research has generally disclosed the existence of homophily for one behavior as related to a single attribute. In this study, I examine the existence and level of homophily as related to a variety of behaviors and attributes. Moreover, revealing such a phenomenon may help us understand how individuals build their networks depending on the attributes of other individuals.

*Macaca* (*Macaca* sp.) societies are characterized by their common social organization, but they are also known for their different social styles. Extensive research has shown that dominance hierarchies vary greatly in the macaque genus (i.e., dominance styles) (De Waal and Luttrell, 1989; Thierry et al., 2000; Sueur et al., 2011). Furthermore, the hierarchical structure of females in the *Macaca* taxon is a well-studied phenomenon that appears to be entirely dependent on the MRI (Thierry et al., 2000). In contrast, each *Macaca* species has stable multi-male, multi-female, and multi-generational social groups in which females are philopatric and males migrate. These common characteristics allow the elucidation of the influence of individual attributes on the interactions between individuals and represents an excellent biological model for this study.

In this study, I use SNA tools to determine individual positions and interactional patterns according to four specific attributes (age, gender, matriline, and hierarchical rank) in affiliative and agonistic networks in *M. sylvanus*. Based on previous studies, several assumptions can be made in response to the following questions:

(1) Who are the most central individuals? As in many cercopithecines, *M. sylvanus* females are the philopatric gender, which should increase their ability to form denser, stronger, and more perennial networks than males (Smuts, 1985). Thus, they can be expected to be the most active and central individuals in the affiliative network. Exploring such functions could reveal the significance of their role in facilitating group cohesion. Males are generally the more aggressive individuals (Gray, 1971), and therefore should be particularly active and central in the agonistic network.
(2) How age and gender influence the positions and roles of individuals? According to our extensive knowledge of the MRI process, we can expect to observe age-related behavioral variations in females that are highly correlated with their reproductive status (Borries et al., 1991; Chapais, 2004) and matriline. The social activity of young females would therefore be more intense (affiliatively and agonistically), with a decrease in activity during their latter ontogenesis, which in some cases may lead to social exclusion at an advanced age. In males, a minimum hierarchical level for older individuals may exist that enables them to maintain a certain ranking (Sosa, 2015). Such kinetics among males reduces their chances of experiencing social exclusion and thus they may face only a minor decrease in social activity, position, and role.
(3) Do common interactional patterns exist among individuals according to their attributes? One sociological model predicts attractiveness to high-ranking females (Seyfarth, 1977). However, this model appears subject to variability. It is mainly observed in despotic societies, and attractiveness to low-ranking individuals has been reported in other species (Schino, 2001; Sueur and Petit, 2008). According to these findings, Seyfarth’s model should not apply to *M. sylvanus*. I also expect to observe homophily related patterns such as allogrooming that target same-gender and same-age individuals and kin (Hirsch et al., 2012). As for agonistic behaviors, it is difficult to form any hypothesis, but heterophily can be suspected.

Responding to these questions allows to reveal how individual attributes and social structure (gender philopatry) produce behavioral divergences that lead to different positions and roles within the group (question 1), how these deviations evolve with the ontogenesis of an individual (question 2), and by which mechanisms individuals interact among themselves (question 3). This multilevel approach allows for a better understanding of how these different levels shape the overall structure of the society in *M. sylvanus*.

## Materials and Methods

### Study Site and Subjects

The current study was conducted over a period of 4 months (July to October 2011) in the park *La Forêt des singes*, in Rocamadour, France. The 141 *M. sylvanus* individuals in the park are divided into three groups and live in semi-free ranging conditions (Sugiyama, 2015) in a 20 hectare forest. They are fed in foraging areas twice per day and have water *ad libitum*. For more details on the management of the park, refer to de Turckheim and Merz (1984). The demographic data (gender, age, and matriline) were provided by the scientific director of the park, Ms. Ellen Merz. The study focused on one of the three groups. Four newborns were excluded from the observations (three males and one female), so that the number of individuals observed was *N* = 52. The group had a balanced gender ratio of 25 females and 27 males, with an age range between 1 and 25 years old. The individuals were previously identified during 1 month through their tattoos. The observations were conducted with the approval of the park management, an agreement that was subject to the specific condition that I would not directly contact nor handle individuals. As I performed simple observations without any type of intervention, I did not require authorization from the French National Advisory Ethics Committee.

### Behavioral Observations

**FIGURE 1:**
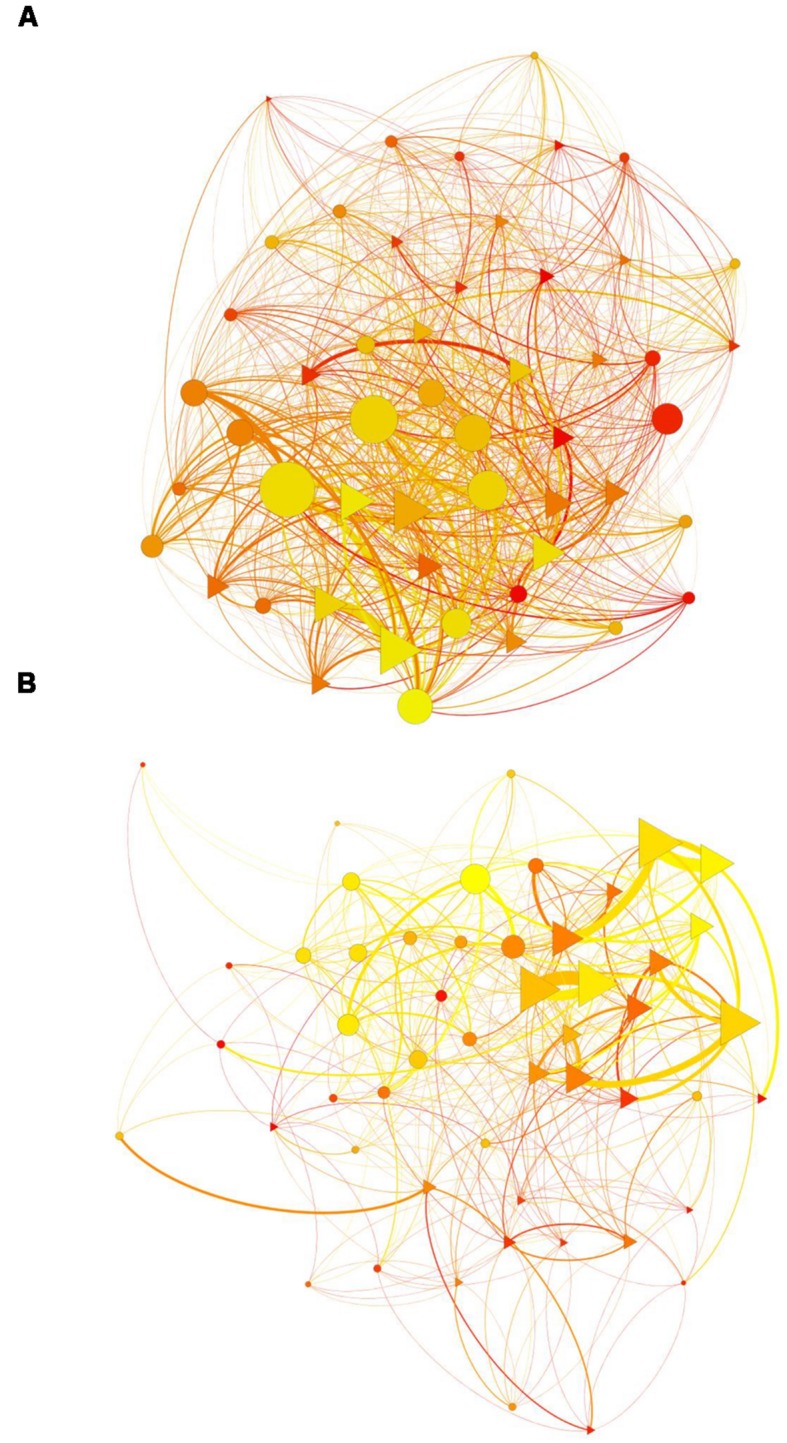
**Social networks: (A) agonistic network, (B) allogrooming network.** Yifan Hu layout. Gradient color of vertices (from yellow to red) represents individuals’ age (from youngest to oldest). Shape of vertices represents individuals’ gender (females: triangles; males: circles). Size of vertices represents individuals’ degree. Size of edges represents the strength of interactions and the color is in accordance with the age of the individual that gives the behavior.

Observations were conducted by repeated focal samplings of 30 min per individual. Each individual was observed approximately 30 times (15 ± 2 h), for 786 observation hours of 52 individuals. Focal sampling time was determined after 2 months of pre-observation. To trade with bias of observation in time of day and feeding time, individuals were observed randomly from 8 am to 5 pm over the 4 months. During the observations, I registered allogrooming and agonistic behaviors (threatening face or growl, charge, avoidance, attack, chase, and aggressive slap, grab, or bite). A complete description of the ethogram of *M. sylvanus* can be found in Hesler and Fischer (2007). An iPad 1 tablet (Inc, 1976) computer and the WhatISee2.0 application (Inc, 2009) were used to register the individuals involved, and the direction, frequency, and duration of the behaviors. Directed and weighted agonistic and allogrooming matrices were built using the obtained behavioral frequencies (**Figure 1**). The overall observation yields a total of 5867 agonistic interactions and 1281 grooming interactions. The agonistic matrices allow us to calculate the hierarchical rank of each individual using David’s Score (David, 1987) with R 3.0.1 (Ihaka and Gentleman, 1996) package steepness (de Vries et al., 2006).

### Social Network Analyses

#### Building Matriline Categories

**FIGURE 2:**
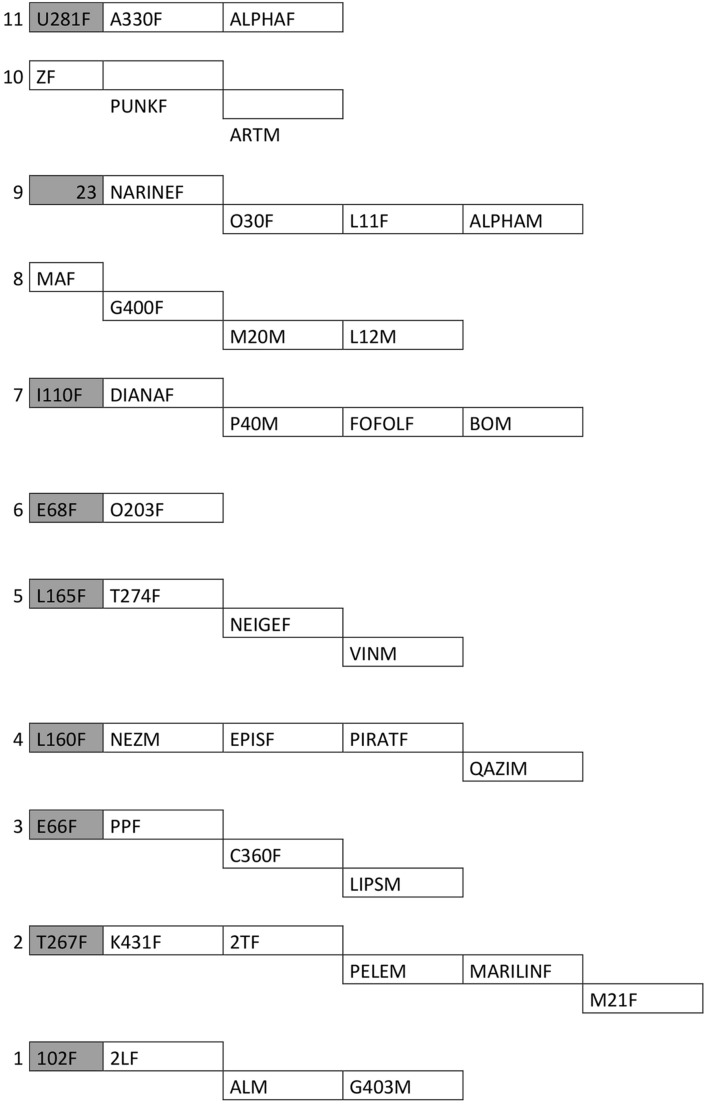
**Matrilines.** This scheme represents individuals which were kept for kinship analysis. Gray cells are dead individuals. Numbers represent the different matrilines. Each subline represents an offspring of the corresponding mother.

Kinship bonds among individuals were determined using two methods. First, data were provided by the park officials who, along with scientists, have been monitoring the population in the park. Second, matrilines were determined through genetic analyses of mitochondrial DNA using eight microsatellite markers. The collection and analysis of DNA samples were performed by the park authorities. The poor quality of DNA samples made some DNA results uncertain. For this reason, matriline groups were built only with individuals whose relatedness was confirmed based on direct observations and genetic analyses. To conserve only close kinship relationships, only the individuals with the same mother were considered related for each mitochondrial haplotype (**Figure 2**). Thus, individuals whose matrilines were uncertain did not belong to any matriline group (eight males and one female). In addition, matriline results must be carefully considered, as not all individuals were taken into account owing to a lack of information on their kinship bonds.

#### Data Consideration before Analysis

Collecting data from all members of the same social group led us to the construction of two social networks (agonistic and affiliative) through the existence of multiple interactions. The intrinsic nature of the collected data (interactions between same-group individuals) underlies the non-independence of the data required by inferential statistical techniques (Wasserman and Faust, 1994; Krause et al., 2009; Brent et al., 2011). Several possibilities exist to deal with this fact. Link filtering is commonly used in animal SNA to delete interactions that can be attributed to random or “chance” events (Croft et al., 2008). However, at present, this filtering process has not been submitted to any formal methodology and has two major limitations: (1) the non-consideration of weak ties (Granovetter, 1973) and thus the loss of important information (Croft et al., 2011); and (2) the sensitivity to data errors such as misidentification.

The approach adopted in this study is based on permutation tests and may help standardize the analysis of animal behavioral data obtained using SNA. In this study, I assumed an approach that allows analysis without the need to filter the links. To this end, the weight of the links must be taken into consideration, which can be done by using weighted social network metrics (Opsahl, 2009; Brent et al., 2011). Frequency-based data are less prone to sampling biases, yet by themselves, they do not solve the issue of data dependency. Therefore, I used weighted network metrics with Null Hypothesis Significant Tests (NHST) involving a permutation-based approach (Manly, 2006). This method generates a set of random values based on the real data set and creates the null hypothesis that the real structural measure X is not different from the random one. This hypothesis is accepted or rejected by comparing the observed value X to the random one. If the observed value is greater than the random one from 95%, then the null hypothesis is rejected. The use of permutation tests in the study of animal societies is discussed in details by Whitehead (2008) and Croft et al. (2011).

The following analyses were performed on both weighted allogrooming and agonistic matrices with 10000 permutations.

#### Network Metrics

For each individual, I calculated the following weighted network metrics: indegree, outdegree, degree, eigenvector centrality, and clustering coefficient with Ucinet 6.375 (Borgatti et al., 2002). Briefly, the degree corresponds to the total number of individuals that directly interact with one given individual (Freeman, 1979). The weighted version takes into account the weight of the links. I also differentiated between indegree (incoming ties) and outdegree (outgoing ties). This metric is historically the first and conceptually the simplest centrality network metric, and in this case, can also be considered as the activity, or “involvement,” of an individual. The eigenvector centrality index is the sum of the connections to neighbors weighted by their degree. This index provides a metric that determines the individual centrality relative to the rest of the network and the “influence” of an individual on the network (i.e., connection to high-degree nodes) and thus, on the social structure. Additionally, it would appear to be a more pertinent centrality metric for non-human primate groups (Kasper and Voelkl, 2009). The weighted clustering coefficient gives weight to the neighborhood densities proportionate to their size and indicates the contribution of each individual in the connectivity and thus, in the cohesion of the network structure (Watts, 2003; Hanneman and Riddle, 2005). For an overview of the weighted network metrics and calculations, see: Wasserman and Faust (1994), Croft et al. (2008) and Whitehead (2008).

### Statistical Analyses

#### Individual Level

For the first analysis, I aimed to study gender, matriline, hierarchical rank, and age-related changes in each network metric. To this end, I used general linear mixed models (GLMM) in which weighted degrees, indegrees, outdegrees, eigenvectors, and clustering coefficients are the dependent variables in separate models. Exact ages, genders, hierarchical ranks, and matrilines are the independent variables.

To offset the non-independence of these data, I realized GLMM with permutation. The consequent biological null hypothesis was that any individual could have any network metric value. Opting for this method has several advantages. First, it takes into account the non-independency of the data; second, it is a better option than multiple *t*-tests and ANOVA (which both need discrete variables and would increase the number of tests) with permutation or simple correlations; and finally, it facilitates the analysis of the interactions between factors.

Initially, I created two types of models: those with no interactions with the dependent variables and those with gender interacting with other individual attributes to examine whether age, matriline, and hierarchical rank dissimilarly influence individuals according to their gender. Only factors estimated higher than 0.009 were considered significant. This threshold is arbitrary and aims to consider only significant effects with sufficient weight. These analyses were performed using SPSS 17 (SPSS, 2008) GLM Procedure with Bootstrap option of *p*-value = 0.05.

#### Group Level

The aim of the second analysis was to examine homophily and heterophily, for which I used NHST (Stephens et al., 2007) with permutation.

The principle of homophily, or the preferential interactions between same-attribute individuals, consequently determines whether the links within a same-attribute group have greater frequencies than the links between groups. Thus, to study homophily between genders, I used a simple *t*-test with permutation for comparing the mean of the links between and within the groups depending on gender.

To study homophily according to age (a continuous attribute), I used the Moran statistic which indexes the differences between the score of an actor and the mean, and then weights the cross products (Moran, 1950). Permutations are used to create a sampling distribution in which scores on the attribute are randomly assigned to actors. As for any permutation test, the real structural measure (the Moran statistic in this case) is compared to the random one (Hanneman and Riddle, 2005). The Moran “I” statistic of autocorrelation ranges from -1.0 (perfect negative correlation) through 0 (no correlation) to +1.0 (perfect positive correlation). These analyses were performed using Ucinet 6.375 (Borgatti et al., 2002).

## Results

### Individual Level

In the agonistic social network, results show that the higher the matriline, the more central (eigenvector: 0.015, *p* < 0.05) and active (degree: 17.866, *p* < 0.05) its members, and the more they receive agonistic behaviors (indegree: 9.857, *p* < 0.05) and contribute to network cohesion (clustering coefficient: 0.077, *p* < 0.01). The results also reveal that the higher the hierarchical rank of an individual, the more it gives agonistic behaviors (outdegree: 6.075, *p* < 0.05), but the less it receives them (indegree: -14.698, *p* < 0.01). Finally, we observe that with age, individuals are less active (degree: -12.529, *p* < 0.01), give less (outdegree: -3.568, *p* < 0.05) and receive fewer (indegree: -8.961, *p* < 0.01) agonistic behaviors. These results are synthetized in **Table 1**.

**Table 1:** General linear mixed models (GLMM) for agonistic network metrics.

GLM with Bootstrap for estimates of fixed effects on agonistic network				
**Network metrics**	**Factor**	**Estimate**	**Standard error**	***p***
Eigenvector	Intercept	0.277	0.065	0.000
	Gender	-0.015	0.026	0.560
	Age	-0.008	0.001	0.000
	**Matriline**	**0.015**	**0.005**	**0.013**
	Hierarchy	-0.006	0.002	0.035
Clustering coefficient	Intercept	1.767	0.464	0.001
	Gender	0.181	0.183	0.330
	**Age**	-**0.037**	**0.009**	**0.001**
	**Matriline**	**0.077**	**0.022**	**0.001**
	Hierarchy	0.021	0.014	0.142
Degree	Intercept	501.027	126.637	0.001
	Gender	-10.413	47.543	0.826
	**Age**	-**12.529**	**2.219**	**0.000**
	**Matriline**	**17.866**	**7.515**	**0.049**
	Hierarchy	-8.624	4.056	0.052
Outdegree	Intercept	-52.915	76.476	0.492
	Gender	13.104	31.082	0.674
	**Age**	-**3.568**	**1.411**	**0.022**
	Matriline	8.010	4.463	0.103
	**Hierarchy**	**6.075**	**2.547**	**0.040**
Indegree	Intercept	553.942	68.172	0.000
	Gender	-23.517	27.990	0.415
	**Age**	-**8.961**	**1.529**	**0.000**
	**Matriline**	**9.857**	**4.129**	**0.039**
	**Hierarchy**	-**14.698**	**2.052**	**0.000**

*In bold, singnificant attributes.*

**FIGURE 3:**
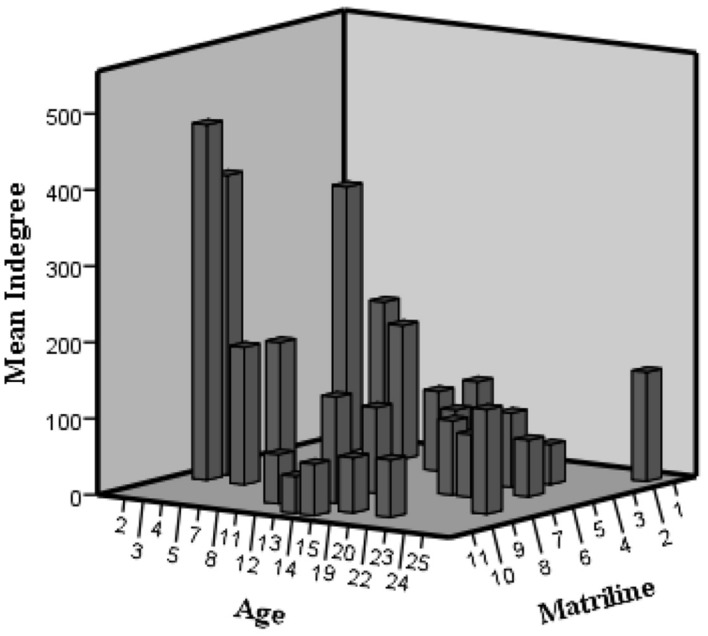
**3D histogram of indegree variation according to age and matriline in the agonistic network**.

The results of the agonistic social network model for gender interactions with other individual attributes are as follows (synthetized in **Table 2**, **Figure 3**, Appendix 2 and 3):

**Table 2:** General linear mixed models for agonistic network metrics for interactions between gender and other individual attributes.

GLM with Bootstrap for estimates of fixed effects on agonistic network				
**Network metrics**	**Factor**	**Estimate**	**Standard error**	***p***
Eigenvector	Intercept	0.281	0.044	0.000
	Males*Age	-0.009	0.040	0.555
	Females*Age	-0.008	0.001	0.000
	Males*Matriline	0.010	0.012	0.305
	Females*Matriline	**0.023**	**0.005**	**0.000**
	Males*Hierarchy	-0.005	0.011	0.393
	Females*Hierarchy	-**0.010**	**0.003**	**0.004**
Clustering coefficient	Intercept	2.300	0.273	0.000
	Males*Age	0.036	0.162	0.706
	Females*Age	-**0.038**	**0.010**	**0.001**
	Males*Matriline	0.059	0.043	0.111
	Females*Matriline	**0.106**	**0.038**	**0.007**
	Males*Hierarchy	-0.008	0.045	0.792
	Females*Hierarchy	0.005	0.022	0.814
Degree	Intercept	529.526	85.390	0.000
	Males*Age	-12.999	42.661	0.512
	Females*Age	-**12.269**	**2.154**	**0.001**
	Males*Matriline	9.245	17.587	0.527
	Females*Matriline	**30.332**	**8.212**	**0.003**
	Males*Hierarchy	-8.301	13.377	0.349
	Females*Hierarchy	-**14.824**	**5.719**	**0.014**
Outdegree	Intercept	-56.542	52.424	0.242
	Males*Age	-15.798	23.689	0.222
	Females*Age	-**3.474**	**1.375**	**0.031**
	Males*Matriline	5.964	10.263	0.468
	Females*Matriline	6.549	5.537	0.201
	Males*Hierarchy	10.129	7.676	0.087
	Females*Hierarchy	**7.989**	**3.708**	**0.047**
Indegree	Intercept	586.068	48.560	0.000
	Males*Age	2.799	23.371	0.786
	Females*Age	-**8.795**	**1.359**	**0.000**
	Males*Matriline	3.281	9.362	0.666
	Females*Matriline	**23.783**	**4.906**	**0.000**
	Males*Hierarchy	-**18.430**	**7.197**	**0.009**
	Females*Hierarchy	-**22.813**	**3.248**	**0.000**

*In bold, significant attributes.*

– The eigenvector model shows that for females, the higher the matriline, the more central the individual (0.023, *p* < 0.01). Furthermore, the centrality of females also decreases with their individual hierarchical rank (-0.010, *p* < 0.01).
– The degree model shows that degree significantly decreases with age for females (-12.269, *p* < 0.01), but not for males. Individual degree increases with matriline (30.332, *p* < 0.01) and decreases with hierarchical rank for females only (-14.824, *p* < 0.05).
– The outdegree model shows that outdegree significantly decreases with age (-3.474, *p* < 0.05) for females only. Individual outdegree increases with hierarchical rank for females (7.989, *p* < 0.05).
– The indegree model shows that indegree significantly decreases with age for females (-8.795, *p* < 0.01). Individual indegree increases with matriline (23.783, *p* < 0.05) for females. However, indegree decreases with hierarchical rank for both males and females (males: -18.430, *p* < 0.01; females: -22.813, *p* < 0.01).
– The clustering coefficient model shows that clustering coefficient significantly decreases with age (-0.038, *p* < 0.01) and increases with matriline (0.106, *p* < 0.01) for females only.

The allogrooming social network is primarily influenced by gender and age, with matriline having no significant effect. We also observe that the higher the hierarchical rank, the more an individual receives allogrooming (indegree: 1.366, *p* < 0.05). Furthermore, with age, individuals are less central (eigenvector: -0.014, *p* < 0.01), less active (degree: -5.625, *p* < 0.01), and give (outdegree: -3.082, *p* < 0.01) and receive (indegree: -2.064, *p* < 0.01) less allogrooming. Interestingly, whereas no significant difference was observed between males and females in the agonistic network, in the allogrooming network, females are more central (eigenvector: 0.231, *p* < 0.01) and more active (degree: 71.708, *p* < 0.01) than males. Allogrooming behaviors are mostly given (outdegree: 32.018, *p* < 0.01) and received by females (indegree: 39.691, *p* < 0.01). These results are synthetized in **Table 3**.

**Table 3:** General linear mixed models for allogrooming network metrics.

GLM with Bootstrap for estimates of fixed effects on allogrooming network				
**Network metrics**	**Factor**	**Estimate**	**Standard error**	***p***
Eigenvector	Intercept	-0.230	0.085	0.015
	**Gender**	**0.231**	**0.045**	**0.001**
	**Age**	-**0.014**	**0.003**	**0.002**
	Matriline	0.003	0.004	0.404
	Hierarchy	0.004	0.003	0.122
Clustering	Intercept	-0.007	0.607	0.990
	Gender	0.681	0.321	0.056
	Age	-0.035	0.032	0.362
	Matriline	0.061	0.045	0.206
	Hierarchy	-0.009	0.027	0.734
Degree	Intercept	-12.075	26.373	0.638
	**Gender**	**71.708**	**14.753**	**0.001**
	**Age**	-**5.625**	**0.947**	**0.000**
	Matriline	1.210	1.675	0.457
	Hierarchy	0.393	0.840	0.624
Outdegree	Intercept	37.450	12.688	0.007
	**Gender**	**32.018**	**7.777**	**0.004**
	**Age**	-**3.560**	**0.475**	**0.000**
	Matriline	0.524	0.907	0.545
	Hierarchy	-0.973	0.425	0.027
Indegree	Intercept	-49.525	18.027	0.011
	**Gender**	**39.691**	**8.388**	**0.001**
	**Age**	-**2.064**	**0.518**	**0.002**
	Matriline	0.686	0.931	0.437
	**Hierarchy**	**1.366**	**0.522**	**0.012**

*In bold, significant attributes.*

The results of the allogrooming social network model for gender interactions with other individual attributes are as follows (synthetized in **Table 4**, Appendix 4 and 5):

**Table 4:** General linear mixed models for allogrooming network metrics for interactions between gender and other individual attributes.

GLM with Bootstrap for estimates of fixed effects on allogrooming network				
**Network metrics**	**Factor**	**Estimate**	**Standard error**	***p***
Eigenvector	Intercept	0.215	0.095	0.036
	Males*Age	0.017	0.018	0.148
	Females*Age	**-0.014**	**0.004**	**0.005**
	Males*Matriline	**-**0.002	0.007	0.722
	Females*Matriline	0.005	0.012	0.669
	Males*Hierarchy	**-**0.011	0.007	0.074
	Females*Hierarchy	0.004	0.007	0.620
Clustering coefficient	Intercept	2.055	0.632	0.019
	Males*Age	0.152	0.129	0.114
	Females*Age	**-**0.024	0.029	0.500
	Males*Matriline	**-**0.031	0.050	0.454
	Females*Matriline	0.230	0.118	0.119
	Males*Hierarchy	**-**0.087	0.050	0.079
	Females*Hierarchy	**-**0.103	0.066	0.187
Degree	Intercept	108.684	24.574	0.001
	Males*Age	2.393	6.459	0.612
	Females*Age	**-5.836**	**1.010**	**0.001**
	Males*Matriline	1.526	2.400	0.495
	Females*Matriline	**-**2.140	3.934	0.583
	Males*Hierarchy	**-**3.576	2.393	0.073
	Females*Hierarchy	2.626	2.048	0.210
Outdegree	Intercept	95.139	11.878	0.000
	Males*Age	2.467	3.206	0.272
	Females*Age	**-3.724**	**0.514**	**0.000**
	Males*Matriline	1.099	1.360	0.382
	Females*Matriline	**-**1.061	2.134	0.607
	Males*Hierarchy	**-**3.593	1.247	**0.002**
	Females*Hierarchy	**-**0.093	1.040	0.927
Indegree	Intercept	13.546	16.413	0.378
	Males*Age	**-**0.075	3.757	0.978
	Females*Age	**-2.112**	**0.579**	**0.003**
	Males*Matriline	0.427	1.203	0.678
	Females*Matriline	**-**1.080	2.201	0.608
	Males*Hierarchy	0.018	1.350	0.989
	Females*Hierarchy	**2.719**	**1.236**	**0.034**

*In bold, significant attributes.*

– The eigenvector model shows that eigenvector significantly decreases with age for females (-0.014, *p* < 0.01), but not for males.
– The degree model shows that degree significantly decreases with age for females (-5.836, *p* < 0.01).
– The outdegree model shows that outdegree significantly decreases with age for females (-3.724, *p* < 0.01).
– The indegree model shows that for females, indegree significantly decreases with age (-2.112, *p* < 0.01) and increases with hierarchical rank (2.719, *p* < 0.05).
– The clustering coefficient model shows non-significant results with any individual attribute.

### Group Level

With respect to agonistic behaviors, we obtain homophily for gender (difference in means: -1.651, *p* < 0.05) and for age (*I* = 0.273, *p* < 0.05). Testing genders separately, we obtain homophily by age for females (*I* = 0.336, *p* < 0.05) and for males (*I* = 0.211, *p* < 0.05). The results for matriline and individual hierarchical rank were non-significant.

For allogrooming, homophily is observable for gender (difference in means: -1.942, *p* < 0.05) and for age (*I* = 0.318, *p* < 0.05). Testing genders separately to analyze if there are homophilic differences between genders according to age, we obtain homophily by age for females (*I* = 0.565, *p* < 0.05), but we do not obtain significant results for males according to age (*I* = 0.100, *p* = 0.106). Homophily is also observed by matriline (*I* = 0.321, *p* < 0.05) Testing genders separately, we observe significant homophily by matriline for females (0.458, *p* < 0.01), but not for males. Finally, we also observe homophily by hierarchical rank (*I* = 0.228, *p* < 0.05), yet testing genders separately, we do not observe significant results in either gender.

## Discussion

In this study, I established an analytical protocol that balances the inter-dependency of the data without filtering the links and that considers the weight of the links, and I analyzed the effects of several factors (gender, age, matriline, and hierarchical rank) at different levels of social organization in a non-human primate species, *M. sylvanus*. These findings reveal to what extent SNA facilitates the investigation of various aspects of animal societies by studying: (1) the position and influence of individuals according to their attributes; (2) the attribute-related network; and (3) the interactional dynamics reflected by homophily. In this way, I demonstrated that the sociogenesis process (rank acquisition) is intimately linked to ontogenesis (i.e., it is age-related), and differs between genders. Hence, individuals with common attributes have similar positions and roles in the group. I also stressed the existence of homophily in several behaviors, reflecting common individual behavioral patterns, including: (1) the acquisition of status within an age-related category, leading to intra-generational conflicts; (2) high-ranking individuals preferably groom similar-rank and opposite-gender individuals to secure better protection and support; and (3) the existence of homophily in grooming behaviors by gender, age, hierarchical rank, and matriline. The results suggest six main findings.

First, we observe that variations in individual attributes have a greater impact on the position, role, and interactional patterns of females than on males. In most cases, these dissimilarities result from the social structure of females in *M. sylvanus* that is based on philopatry and MRI. Additionally, we observe significant disparities in activity and centrality between males and females. Females are more central and active (for both received and given behaviors) in the allogrooming network. More specifically, they give and receive more allogrooming, mainly with individuals who have similar characteristics, namely females according to homophily results. These findings are in line with the literature that stresses that the philopatric gender plays a key role in affiliative behaviors (Aureli and de Waal, 2000; Silk, 2001). From a biological perspective, the philopatric gender has more time to develop a denser, stronger, and perennial network than the non-philopatric gender. In addition, female matriline homophily results emphasize the relevance of kinship bonds among females in affiliative behaviors. Individuals with high centrality and activity thus preferentially contribute to the establishment of the global network structure (Lusseau and Newman, 2004; Sosa, 2014) and cohesion of the group. In *M. sylvanus*, these key individuals are unquestionably the females.

Second, we observe that for female *M. sylvanus*, network metrics decrease with age in the agonistic and allogrooming networks. In the agonistic network, older females are less active (degree, indegree, and outdegree) and less involved in the cohesion of the network (clustering coefficient). In the allogrooming network, older females are less active (degree, indegree, and outdegree) and less central (eigenvector). During the early years, high centrality and activity for allogrooming behaviors is likely related to a long period of mother–infant and kin-related preferential interactions (which is supported by the results of matriline homophily for allogrooming behaviors) that generates kin recognition and later, kin-biased affiliative interactions (Pereira and Fairbanks, 1993). Furthermore, juveniles learn how to interact by relating to their close relatives. The observed decrease of allogrooming centrality and activity with age is likely related to the progressive stabilization of the affiliative networks of females. In addition, the decline with progressing age in the agonistic network (as related to activity and the role in the cohesion of the network) is probably a result of the stabilization of the hierarchical ranks of females when sexual maturity is attained (Chapais, 2004). These results show that the sociogenesis process, or rank acquisition, is intimately linked to ontogenesis (i.e., it is age-related), with the latter being closely related to the reproductive status of females (menarche and postmenopause) (Borries et al., 1991). This ontogenetic process can be characterized into three stages. The first occurs before sexual maturity, when the female has numerous social interactions in order to establish her position within the group. Second, once the female is mature, she has fewer social interactions, which indicates a period of stabilization of her position. The final stage corresponds to the postmenopausal period, which can lead to even fewer social interactions resulting from exclusion (Borries et al., 1991; Sosa, 2015). Unlike females, males are not subject to the phenomenon of declining social interactions, as none of their network metrics significantly decreases with age.

Third, the frequency of given agonistic behaviors increases with the hierarchical rank of a female. More specifically, the higher her hierarchical position, the greater number of submissive individuals with whom to ensure her rank a female has (Tokuda and Jensen, 1969) and the more she intervenes in conflicts to provide support (De Waal and Roosmalen, 1979; Seyfarth and Cheney, 1984; De Waal, 1997). This does not appear to be a response to received agonistic behaviors as the indegree would also increase, which is not the case. Instead, the agonistic indegree, together with the eigenvector, decreases with the hierarchical rank of a female. This decline in agonistic indegree is also observed in males, stressing that high-ranking individuals, regardless of gender, receive fewer agonistic behaviors than low-ranking ones. This reveals the benefits of dominant positions, with the reduction of associated risks (Gartlan, 1968; Bernstein, 1976; Chapais, 1991). Additionally, a significant relationship between the frequency of received allogrooming behaviors and hierarchical rank is observed in females. The absence of such phenomenon among males can be attributed to the fact that attractiveness to high-ranking individuals in allogrooming for males seems species-specific (Watts, 2000). Nonetheless, this phenomenon is observed among females according to GLMM results, which is in accordance with the theory advanced by Seyfarth (1977) in which dominant females should be preferred allogrooming partners as they can provide better protection (Watts, 2000; Cheney and Seyfarth, 2008) and support (Cheney and Seyfarth, 1990).

The fourth finding is that, similar to the hierarchy results, females within the same matriline have similar centralities and activities, and the higher the matriline, the more central and active the female is in the agonistic network. This trend can be attributed to the fact that in the genus *Macaca*, the hierarchical ranks of females are intimately linked to their matrilines. Furthermore, a closer examination of the previously discussed high agonistic activity of immature females shows that the indegree is more intense for high-born ones (**Figure 3**). Before sexual maturity, females must settle their dominance relations with lower matriline-ranking females through agonistic interactions (Chapais (2004). Thus, in their early years, females compete to establish their hierarchical rank on multiple fronts: (1) within their own matriline (as supported by the MRI phenomenon); (2) within their age category (in accordance with age homophily results for agonistic behaviors); and (3) toward older lower-ranking females.

Fifth point is that homophily results for the agonistic network show that agonistic interactions are mainly directed within the same age and gender. These findings, combined with GLMM results, yield interesting biological interpretations. Higher activity and connections in the agonistic ego network among young individuals (GLMM results) can be interpreted as a phenomenon of hierarchical rank acquisition. Homophily results highlight that this rank acquisition occurs mainly between same-age and same-gender individuals (Chapais, 1988; Holekamp and Smale, 1991), stressing the existence of a particular phenomenon that we could call the intra-generational conflict. Furthermore, the fact that affiliative behaviors are also primarily directed toward same-age individuals (age homophily results for allogrooming behaviors) underlines the trend of individuals to build their affiliative network within their age category.

Sixth, we observe homophily in allogrooming behaviors according to hierarchical rank, but only when both genders are taken into account. In other words, opposite-gender individuals with similar hierarchical ranks have preferential affiliative interactions. Allogrooming behaviors facilitate the creation of affiliative bonds and potential support in future conflicts (De Waal and Roosmalen, 1979; Seyfarth and Cheney, 1984; De Waal, 1997). Subsequently, this behavioral pattern can explain the decrease of agonistic behaviors received by high-ranking individuals (observed in GLMM results) owing to the high-ranking support of a third party.

Homophily has previously been reported in many species (McPherson et al., 2001; Lusseau and Newman, 2004; Massen and Koski, 2013). Extensive research on human homophily stressed that it is a major mechanism in stranger cooperation (Haun and Over, 2015), social learning (Buttelmann et al., 2013), and cultural and norms transmission (Chudek and Henrich, 2011). Recent studies argue that homophilic preferences may explain the gap between animals and humans regarding these abilities (Haun and Over, 2015). Revealing homophily in several behaviors and as it is influenced by different attributes highlights the importance of these mechanisms in a non-human primate species. However, many methods exist to evaluate the presence or absence of homophily (E-I index, ERGM, assortativity, Moran I statistic), each one of them with inherent pros and cons that would need to be evaluated before determining which of these approaches is more relevant for studying animal societies.

This analytical protocol can be used to study other animal societies and might enable interspecific comparisons. I believe that the important findings of this study might help understand the global patterning of a non-human primate society, and likely other animal societies, from an evolutionary perspective.

## Author Contributions

The author confirms being the sole contributor of this work and approved it for publication.

## Conflict of Interest Statement

The author declares that the research was conducted in the absence of any commercial or financial relationships that could be construed as a potential conflict of interest.
