# Supplementary material for: The Influence of Gender, Age, Matriline and Hierarchical Rank on Individual Social Position, Role and Interactional Patterns in Macaca sylvanus at ‘La Forêt des Singes’: A Multilevel Social Network Approach
Source: Front Psychol. 2016 Apr 18;7:529. doi: 10.3389/fpsyg.2016.00529 (PMC4834345; doi:10.3389/fpsyg.2016.00529)
Supplement: Supplementary file 4 [file Image_4.PDF]

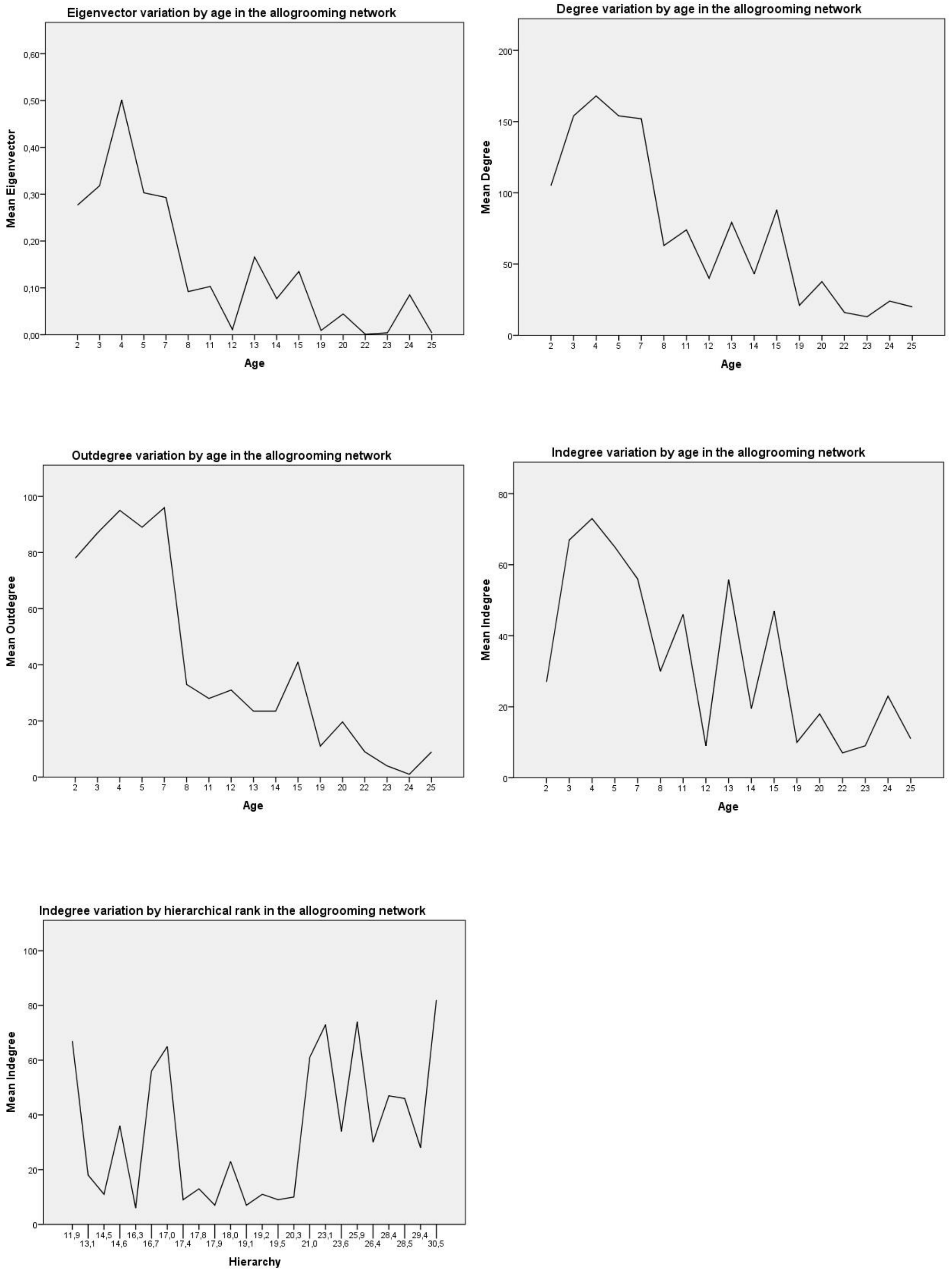

Appendix 4. *Females' allogrooming network metrics: significant variation according to individual attributes.*
